# Impact of creatine supplementation on menopausal women’s body composition, cognition, estrogen, strength, and sleep

**DOI:** 10.1080/15502783.2025.2533673

**Published:** 2025-07-16

**Authors:** Lauren Hall, Sadie Klassen, Jennifer Holbein, John Waters

**Affiliations:** St. Olaf College, Kinesiology Department, Northfield, Minnesota, USA

**Keywords:** Menopause, cognition, body composition, sleep, estradiol, strength, creatine, creatine supplementation, estrogen

## Abstract

**Background:**

Despite extensive research on creatine, evidence for use among females is understudied. Understanding the effects of creatine metabolism in peri- and post menopause yields important implications for creatine supplementation regarding performance and health in females. This repeated-measures quasi-experimental study analyzes the effects of Creatine Monohydrate on performance, body composition, cognitive function, estrogen levels, mood and sleep of females in perimenopause and postmenopause.

**Methods:**

TFifteen females with a mean age of 54 with 5 in perimenopause and 10 in postmenopause participated in this 14 week study. The participants in each group attended 9 sessions over the 14 weeks, following a twice-weekly training schedule based total body strength training program written by the college conditioning coach. The study used a Bod Pod® to analyze body composition, an estradiol spit test to observe estrogen levels, cognitive function assessments, a weekly mood and sleep questionnaire, and an isokinetic dynamometer to examine muscle strength.

**Results:**

Creatine supplementation led to significant increases in lower body strength across peri- and postmenopausal participants, particularly in the test of the isometric concentric extensor peak torque at 60° for 3 seconds (p < .05). Perimenopausal women demonstrated positive improvements in sleep quality (p = .0181). There were no significant results observed in estradiol levels or non-Humac tests.

